# Evaluation of a newly implemented crisis-resolution and home-treatment team in munich – a mixed-methods-analysis

**DOI:** 10.1192/j.eurpsy.2021.983

**Published:** 2021-08-13

**Authors:** J. Boyens, P. Brieger, J. Hamann

**Affiliations:** 1 Klinik Und Poliklinik Für Psychiatrie Und Psychotherapie, Technical University of Munich, München, Germany; 2 Direction, kbo-Isar-Amper-Klinikum, Haar, Germany

**Keywords:** integrated care, crisis resolution and home treatment, Germany

## Abstract

**Introduction:**

Challenged by the lack of collaboration between treatment sectors in psychiatric care in Germany, a legal basis for the implementation of Stationsäquivalente Behandlung (StäB), a programme for crisis resolution and home treatment (CRHT), was formed in 2017. It offers intensive care to patients with severe mental illness in their own living environments, carried out by a team of diverse professionals.

**Objectives:**

The present analysis is the first to evaluate the CRHT-program that has been established in the greater Munich area in 2018.

**Methods:**

Qualitative and quantitative data were collected within the framework of a mixed-methods-analysis. Records of all patients (N=139) included in the CRHT over a thirteen-month period (’18–’19) were examined regarding sociodemographic, clinical parameters, and treatment data. A focus group with StäB-employees (N=8) and individual interviews with patients (N=10) were conducted, then transcribed, and analysed using thematic analysis.

**Results:**

139 patients (74% female) were treated in 164 cases for 38 days on average. Main diagnoses were schizophrenic diseases (43%) and mood disorders (35%), with patients ranging from markedly to severely ill (mean CGI-S: 5.8). 9.4% were in postpartum. Qualitative analysis is still in progress. Preliminary results demonstrate positive responses to individual treatment and environmental integration, whereas frequently changing contacts and the logistical effort were seen critically.

**Conclusions:**

Work is still in progress. We expect StäB to be an adequate alternative to inpatient treatment for women in puerperium and a new opportunity for patients who need intensive treatment but refuse hospitalisation.

